# The risk of miscarriage following COVID-19 vaccination: a systematic review and meta-analysis

**DOI:** 10.1093/humrep/dead036

**Published:** 2023-02-16

**Authors:** Michael P Rimmer, Jhia J Teh, Scott C Mackenzie, Bassel H Al Wattar

**Affiliations:** Medical Research Council Centre for Reproductive Health, Institute of Regeneration and Repair, Edinburgh BioQuarter, University of Edinburgh, UK; Faculty of Medicine, Department of Metabolism, Digestion and Reproduction, Imperial College London, London, UK; Medical Research Council Centre for Reproductive Health, Institute of Regeneration and Repair, Edinburgh BioQuarter, University of Edinburgh, UK; Beginnings Assisted Conception Unit, Epson and St Helier University Hospitals, London, UK; Comprehensive Clinical Trials Unit, Institute for Clinical Trials and Methodology, University College London, London, UK

**Keywords:** COVID-19 vaccination, vaccine safety, miscarriage, pregnancy loss, live birth

## Abstract

**STUDY QUESTION:**

What is the risk of miscarriage among pregnant women who received any of the COVID-19 vaccines?

**SUMMARY ANSWER:**

There is no evidence that COVID-19 vaccines are associated with an increased risk of miscarriage.

**WHAT IS KNOWN ALREADY:**

In response to the COVID-19 pandemic, the mass roll-out of vaccines helped to boost herd immunity and reduced hospital admissions, morbidity, and mortality. Still, many were concerned about the safety of vaccines for pregnancy, which may have limited their uptake among pregnant women and those planning a pregnancy.

**STUDY DESIGN, SIZE, DURATION:**

For this systematic review and meta-analysis, we searched MEDLINE, EMBASE, and Cochrane CENTRAL from inception until June 2022 using a combination of keywords and MeSH terms.

**PARTICIPANTS/MATERIALS, SETTING, METHODS:**

We included observational and interventional studies that enrolled pregnant women and evaluated any of the available COVID-19 vaccines compared to placebo or no vaccination. We primarily reported on miscarriage in addition to ongoing pregnancy and/or live birth.

**MAIN RESULTS AND THE ROLE OF CHANCE:**

We included data from 21 studies (5 randomized trials and 16 observational studies) reporting on 149 685 women. The pooled rate of miscarriage among women who received a COVID-19 vaccine was 9% (n = 14 749/123 185, 95% CI 0.05–0.14). Compared to those who received a placebo or no vaccination, women who received a COVID-19 vaccine did not have a higher risk of miscarriage (risk ratio (RR) 1.07, 95% CI 0.89–1.28, *I*^2^ 35.8%) and had comparable rates for ongoing pregnancy or live birth (RR 1.00, 95% CI 0.97–1.03, *I*^2^ 10.72%).

**LIMITATIONS, REASONS FOR CAUTION:**

Our analysis was limited to observational evidence with varied reporting, high heterogeneity and risk of bias across included studies, which may limit the generalizability and confidence in our findings.

**WIDER IMPLICATIONS OF THE FINDINGS:**

COVID-19 vaccines are not associated with an increase in the risk of miscarriage or reduced rates of ongoing pregnancy or live birth among women of reproductive age. The current evidence remains limited and larger population studies are needed to further evaluate the effectiveness and safety of COVID-19 vaccination in pregnancy.

**STUDY FUNDING/COMPETING INTEREST(S):**

No direct funding was provided to support this work. M.P.R. was funded by the Medical Research Council Centre for Reproductive Health Grant No: MR/N022556/1. B.H.A.W. hold a personal development award from the National Institute of Health Research in the UK. All authors declare no conflict of interest.

**REGISTRATION NUMBER:**

CRD42021289098.

## Introduction

The last 2 years saw the mass rollout of multi-national vaccination campaigns for the SARS-CoV-2 (COVID-19) virus with the hope of attenuating its devastating effect on society and restoring normality (de Gier *et al.*, 2021; Lopez Bernal *et al.*, 2021). The rapid development and rollout of these vaccines raised concerns about their short- and long-term health side effects leading to vaccine hesitancy among pregnant women and those planning a pregnancy (Egloff *et al.*, 2022; Kiefer *et al.*, 2022). However, to date, most studies and regulatory bodies support their safety and effectiveness (American College of Obstetricians and Gynecologists, 2022; Royal College of Obstetricians & Gynaecologists, 2022; UK Health Security Agency, 2022). Most early studies evaluating the efficacy of COVID-19 vaccines excluded pregnant women, which limited evidence synthesis on the safety of vaccines in pregnancy (Polack *et al.*, 2020; Baden *et al.*, 2021; Madhi *et al.*, 2021; Sadoff *et al.*, 2021). The majority of health authorities currently support the safety of COVID-19 vaccination in pregnant women (American College of Obstetricians and Gynecologists, 2022; Royal College of Obstetricians & Gynaecologists, 2022) to reduce the risk of poor pregnancy outcomes observed in unvaccinated women with COVID-19 infection (Stock *et al.*, 2022).

Some authors have raised concerns about the potential cross-reactivity of SARS-CoV-2 spike protein antibodies following mRNA vaccination with human syncytin-1 protein in trophoblastic tissue (Ciapponi et al., 2021; Mattar *et al.*, 2021; Shanes *et al.*, 2021; Schaler and Wingfield, 2022). Autoreactive antibodies against syncytin-1 were presumed to cause placental damage and early pregnancy loss due to the potential homology with the SARS-CoV-2 spike protein. However, further characterization of the SARS-CoV-2 spike protein structure and amino acid sequencing showed low homology with syncytin-1, disproving claims of cross-reactivity, and potential damage to placental tissue (Gong *et al.*, 2005; Kloc *et al.*, 2021; Prasad *et al.*, 2021). Given the increased risk of morbidity and mortality among pregnant women with COVID-19, it is critical to maximize prevention efforts by encouraging vaccine uptake and promoting its safety during pregnancy (Royal College of Obstetricians & Gynaecologists, 2021). We performed a systematic review and meta-analysis of the available literature to evaluate the rates of miscarriage and live birth among women who received a COVID-19 vaccination.

## Materials and methods

We performed a systematic review and meta-analysis using a prospectively registered protocol (CRD42021289098) and reported our findings as per PRISMA guidelines (Page *et al.*, 2021).

### Search strategy

We searched MEDLINE, EMBASE, and Cochrane CENTRAL until June 2022 using a combination of keyword and MeSH terms for studies of any design that compared the risk of miscarriage and other pregnancy outcomes between vaccinated and non-vaccinated pregnant women (Supplementary Data File S1).

### Study selection and inclusion process

Relevant studies were screened in duplicate (M.P.R. and J.J.T.). Studies of any design that reported on miscarriage and other pregnancy outcomes in women who received any COVID-19 vaccine with or without a control cohort (placebo or no vaccine) were included. We excluded animal studies, those reporting on non-clinical outcomes in human participants, review articles and case reports. Data submitted to health regulators for evaluation of vaccine effectiveness and safety were also included if they were made publicly available ahead of peer review.

### Data extraction

Data extraction was performed in triplicate (M.P.R., J.J.T., and S.C.M.) using a piloted electronic data collection tool with the following characteristics collected: study publication year and journal, inclusion–exclusion criteria, type of intervention and comparison evaluated, characteristics of the included study population and the evaluated COVID-19 vaccine, and all relevant clinical outcomes.

### Outcome measures

We reported on the following pregnancy outcomes: miscarriage (defined as spontaneous loss of a pregnancy before 24 weeks gestation), live birth (defined as the birth of a live child after 24 weeks gestation), and ongoing pregnancy (defined as a viable pregnancy after 12 weeks gestation).

### Risk of bias assessment

Two reviewers (M.P.R. and J.J.T.) assessed the risk of bias and applicability of included studies independently using The Risk Of Bias In Non-randomized Studies of Interventions (ROBINS-I) tool (Sterne *et al.*, 2016). We evaluated the risk of bias in the included studies compared to a target randomized trial that evaluated the risk of miscarriage, live birth, and ongoing pregnancy in women of reproductive age who received a COVID-19 vaccine compared to placebo or no vaccine. As most of the included studies were cohorts or interrupted time series that followed up on women who received a COVID-19 vaccine, we assessed the risk of bias due to confounding, participant selection, classification of intervention, deviation from the intended intervention, missing data, outcome measurement, and selection of reported results. We then generated an overall risk of bias assessment for each study. Studies were deemed to be low risk of bias if they were assessed as low risk in all domains, moderate risk of bias if they were assessed as low or moderate risk of bias in any domain, serious risk of bias if they were assessed as serious risk of bias in at least one domain, but not at critical risk of bias in any domain, or critical risk of bias if one or more domains was assessed as critical.

### Statistical analysis

We pooled data to evaluate the overall rate of miscarriage and live birth/ongoing pregnancy across all women who received a COVID-19 vaccine and generated a pooled risk ratio (RR) compared to women who were not vaccinated. We reported on the pooled event rate using risk with 95% CIs. For our comparative meta-analysis, we reported on dichotomous outcomes using summary RR with 95% CI and on continuous outcomes using weighted mean difference with 95% CI. We used a random effect meta-analysis and applied a restricted maximum likelihood model. Study heterogeneity among included trials was assessed using the *I*^2^ statistic. We also assessed the publication bias and small study effect using a funnel plot for each pairwise comparison and performed Egger’s test to assess its statistical significance. We planned a sensitivity meta-regression and subgroup analyses to investigate potential effect modifiers where relevant. All statistical analyses were conducted in Stata V13 (StataCorp, TX, USA) and Open Meta-analyst software (Brown University, Providence, RI, USA).

## Results

**Figure 1.**
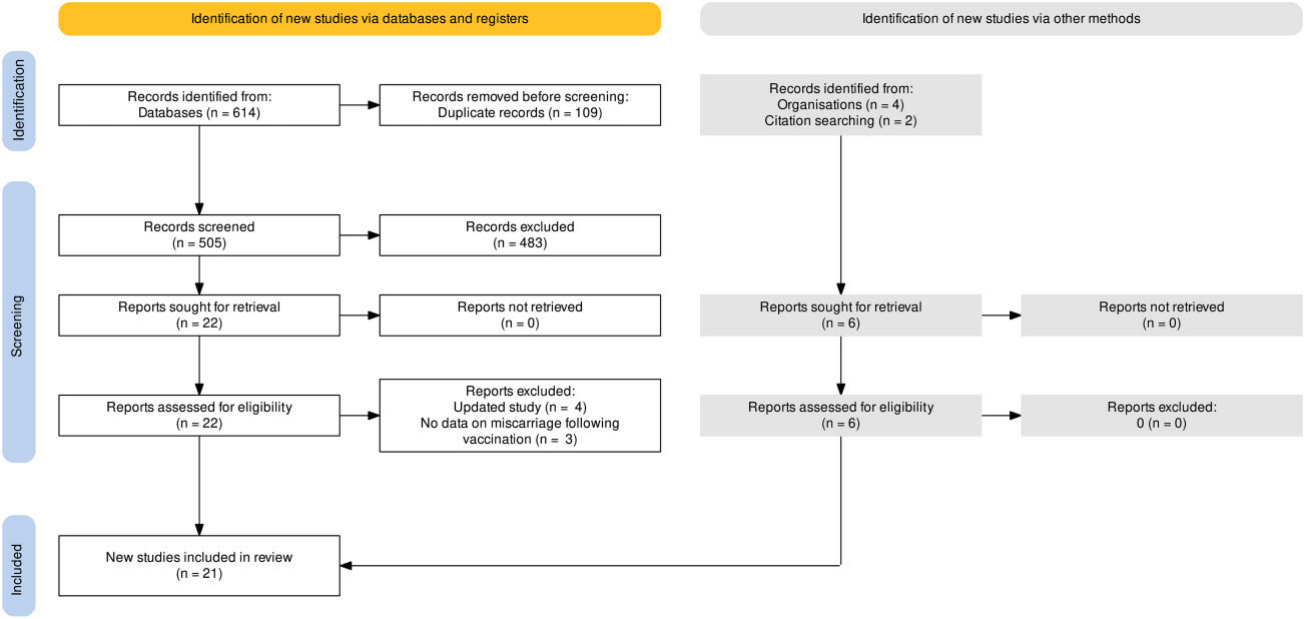
**Study screening and inclusion process for systematic review evaluating the risk of miscarriage and ongoing pregnancy live birth among pregnancy women who received COVID-19 vaccine**.

We screened 505 potentially relevant citations, assessed 28 in full and included 21 studies: 5 randomized control trials (RCTs) (United States Food and Drug Administration, 2020a,b, 2021a,b; Hillson *et al.*, 2021) and 16 observational studies (Bleicher *et al.*, 2021; Bookstein Peretz *et al.*, 2021; Kachikis *et al.*, 2021; Kharbanda *et al.*, 2021; Magnus *et al.*, 2021; Nabila Arfah and Murizah, 2021; Qiao *et al.*, 2021; Trostle *et al.*, 2021; Zauche *et al.*, 2021; Aharon *et al.*, 2022; Avraham *et al.*, 2022; Citu *et al.*, 2022; Favre *et al.*, 2022; Huang *et al.*, 2022; Moro *et al.*, 2022; Wang *et al.*, 2022). All together the studies reported on pregnancy outcomes in 149 685 women (Table I and Supplementary Table SI). Two studies reported on the same population (Aharon *et al.*, 2021, 2022), while an additional two studies reported on the same data registry (Shimabukuro *et al.*, 2021; Moro *et al.*, 2022) (Fig. 1). All of the RCTs in this review excluded pregnant women at the time of recruitment but reported on those who became pregnant during the trial.

**Table I:** Characteristics of included studies that evaluated the risk of miscarriage and rates of ongoing pregnancy/live birth among pregnant women who received a COVID-19 vaccine.

Study	Design	Countries	Funding source	Covid-19 vaccine	Vaccine doses	Inclusion criteria	Numbers analysed (n=)	Risk of bias
Aharon (2022)	Cohort	USA	Not stated	Pfizer, Moderna	2	Women undergoing fertility treatment who were vaccinated at least 14 days prior to starting medication for ovarian stimulation or a frozen-thawed embryo transfer cycle	2153	Moderate
Avraham (2022)	Cohort	Israel	No external funding	Pfizer	2	Women 20–42 years old undergoing IVF treatment cycles at a single centre	400	Moderate
Bleicher (2021)	Cohort	USA	Not stated	Pfizer	≥1	Being pregnant at enrolment and valid questionnaire	326	Serious
Bookstein Peretz (2021)	Case-control	Israel	Not stated	Pfizer	2	Pregnant women between 2 and 40 weeks’ gestation who completed two doses of vaccine	57	Serious
Citu (2022)	Cohort	Romania	No external funding	Pfizer, Moderna	≥1	Women aged >18 years who were vaccinated during the first trimester of pregnancy	3094	Moderate
Favre (2022)	Cohort	Switzerland	Swiss Federal Office of Public Health and the CHUV Foundation	Pfizer, Moderna	≥1	Pregnant women with at least one injection between 1 week before last menstrual period to end of pregnancy	228	Moderate
FDA—Janssen (2021)	RCT	Brazil, Chile, Argentine, Colombia, Peru, Mexica, USA, South Africa	Janssen Research and Development	Janssen	1	Adults 18–59 years of age and 60 years of age or older, respectively, who were in good or stable health and did not have coexisting conditions that have been associated with an increased risk of severe COVID-19	8	Low
FDA—Moderna (2020)	RCT	USA	Biomedical Advanced Research and Development Authority and the National Institute of Allergy and Infectious Diseases	Moderna	2	18 years old and had no known history of SARS-CoV-2 infection and whose locations or circumstances put them at appreciable risk of acquiring SARS-CoV-2 infection or who were at high risk for severe disease (or both)	13	Low
FDA—Moderna (Booster) (2021)	RCT	USA	Not stated	Moderna booster	2 + 1	Individuals 65 years of age and older, individuals 18 through 64 years of age at high risk of severe COVID-19, and individuals 18 through 64 years of age whose recent institutional or occupational exposure to SARS-CoV-2 puts them at high risk of serious complications of COVID-19 including severe COVID-19	1	Low
FDA—Pfizer (2020)	RCT	USA, Brazil, Argentina, Turkey South Africa, Germany	BioNTech and Pfizer	Pfizer	2	Adults 16 years of age or older who were healthy or had stable chronic medical conditions, including but not limited to human immunodeficiency virus (HIV), hepatitis B virus or hepatitis C virus infection	23	Low
Hillson (2021)	RCT	UK, Brazil, South Africa	UK Research and Innovation, National Institutes of Health Research (NIHR), The Coalition for Epidemic Preparedness Innovations, the Bill & Melinda Gates Foundation, the Lemann Foundation, Rede D’Or, the Brava and Telles Foundation, NIHR Oxford Biomedical Research Centre, Thames Valley and South Midland's NIHR Clinical Research Network, and AstraZeneca.	AstraZeneca	2	Women enrolled on a RCT, who were thought not to be pregnant but found to be pregnant, and this occurred in four ongoing Phase 1, Phase 2, and Phase 3 clinical trials	67	Low
Huang (2022)	Cohort	China	National Natural Science Foundation of China, Key Research and Development Program of Jiangxi Province	Sinopharm, Sinovac	2	Women undergoing a fresh IVF cycle who had received at least two vaccine doses at least 3 weeks apart	2185	Moderate
Kachikis (2021)	Cohort	USA	National Institute of Child Health and Human Development	Pfizer, Moderna, Janssen	2	Women who were pregnant, lactating, or planning pregnancy at the time of COVID-19 vaccination	6244	Serious
Kharbanda (2021)	Cohort	USA	Centre for Disease Control and Prevention	Pfizer, Moderna, Janssen	≥1	Women with ongoing pregnancies between 6- and 19-weeks’ gestation	105 446	Moderate
Magnus (2021)	Case-control	Norway	Research Council of Norway	Pfizer, Moderna, AstraZeneca	≥1	Women who had miscarriage before 14 weeks of gestation or primary care–based confirmation of ongoing pregnancy in the first trimester	18 477	Low
Moro (2022)	Cohort	USA	No funding received	Pfizer, Moderna, Janssen	≥1	Pregnant women who received COVID-19 vaccine and reported an adverse events to VAERS by using a pregnancy-status question in the form	3462	Moderate
Nabila Arfah (2021)	Cohort	Malaysia	Not stated	mRNA COVID-19 vaccine	≥1	Pregnant women after receiving a mRNA COVID-19 vaccine	45	Serious
Qiao (2021)	Cohort	Brazil	Sinovac Life Sciences	Sinovac, Janssen, AstraZeneca, Pfizer	≥1	Pregnant or postpartum women who reported vaccine-related adverse effects to adverse events following immunization surveillance information system	3333	Moderate
Trostle (2021)	Cohort	USA	Not stated	Pfizer, Moderna	≥1	Pregnant women who received at least one dose of an mRNA COVID-19 vaccination during pregnancy	424	Moderate
Wang (2022)	Cohort	China	National Natural Science Foundation of China	Inactivated COVID-19 vaccine	^2^	Participants who had completed gamete retrieval and embryo cryopreservation prior to vaccination with two doses of inactivated COVID-19 vaccine followed by a frozen-thaw embryo transfer cycle	1496	Moderate
Zauche (2021)	Cohort	USA	Not stated	Pfizer, Moderna	≥1	Singleton pregnancy who had received at least one dose of an mRNA Covid-19 vaccine either before conception or before 20 weeks of gestation and who did not have a pregnancy loss before 6 weeks of gestation	2203	Moderate

Six vaccines were used in included studies, including Pfizer-BioNTech BNT162b2 mRNA, Moderna mRNA-1273 SARS-CoV-2, Janssen Ad26.COV2.S, AstraZeneca ChAdOx1 nCoV-19, Sinopharm BBIBP-CorV, and Sinovac-CoronaVac. Ten studies reported on pregnancy outcomes following at least one vaccine dose, eight studies reported pregnancy outcomes following two doses and one study reported outcomes after a third booster dose (Table I).

### Quality of included studies and risk of publication bias

Overall, the quality of the included studies was considered to have low to moderate risk of bias while four studies were considered to have a serious risk of bias (Supplementary Fig. S1). All included studies were assessed as having missing information on adherence to the vaccine administration schedule, not allowing accurate assessment of the risk of bias for deviations from the intended intervention. Six of the included studies had an overall low risk of bias (6/21, 29%), half showed a moderate risk (11/21, 52%), and 4 showed a high risk of bias (4/21, 19%) mainly due to participant selection and measurement and outcomes reporting. Outcome reporting was poor overall with only two studies offering a clear outcome definitions for miscarriage and ongoing pregnancy (Aharon *et al.*, 2021; Hillson *et al.*, 2021). Our funnel plot suggested no major variation across included studies with a non-significant Egger’s test at *P* = 0.81 (Supplementary Fig. S2).

### Pregnancy outcomes

**Figure 2.**
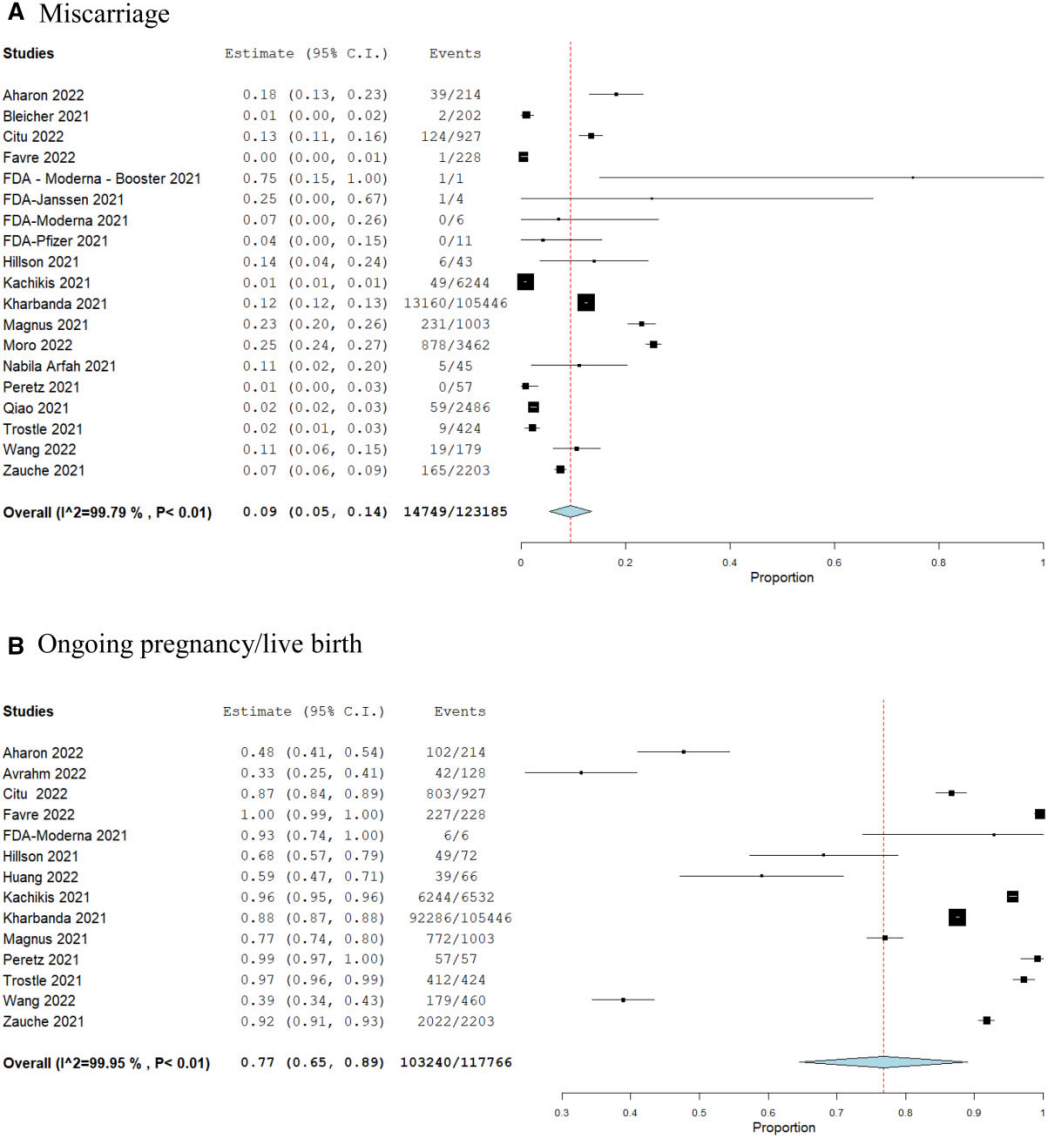
**Pooled event rate of miscarriage and ongoing pregnancy/live birth among pregnancy women who received the COVID-19 vaccinations.** (**A**) Miscarriage. (**B**) Ongoing pregnancy/live birth.

**Figure 3.**
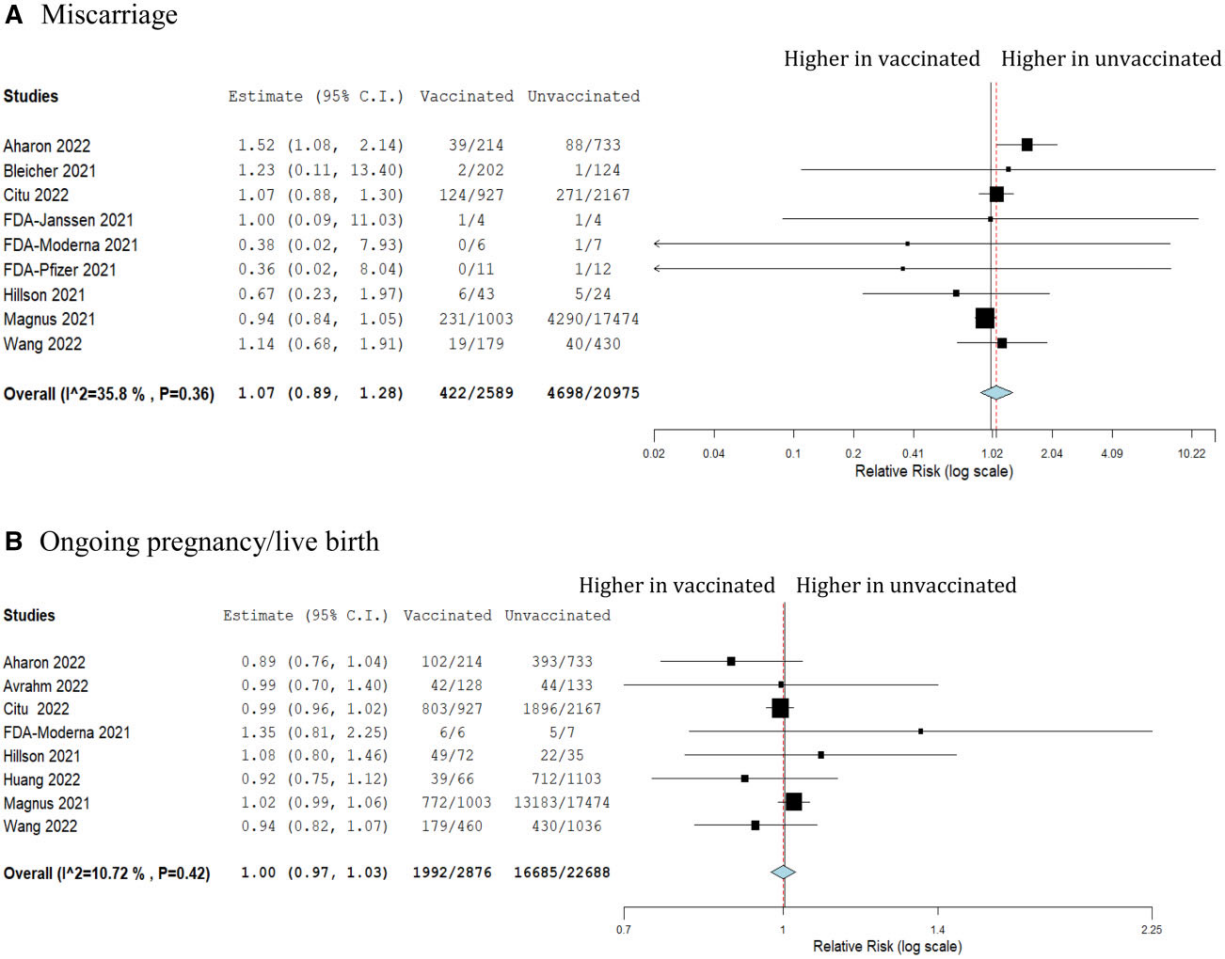
**Forest plot showing the risk ratio of miscarriage and ongoing pregnancy/live birth among pregnancy women who received COVID-19 vaccination compared to unvaccinated women.** (**A**) Miscarriage. (**B**) Ongoing pregnancy/live birth.

We pooled the overall miscarriage rate across all included studies among women who received any COVID-19 vaccine which was 9% (n = 18 studies, 14 749/123 185, 95% CI 0.05–0.14) (Fig. 2). We then compared the risk of miscarriage among those who received any COVID-19 vaccine to those who did not, which suggested no significant difference between the two groups (RR 1.07, 95% CI 0.89–1.28, *I*^2^ 35.8%) (Fig. 3).

The overall proportion of women with ongoing pregnancies or live birth among those who were vaccinated was consistent with the reported population levels at 77% (n = 14 studies, 103 240/117 766, 95% CI 0.65–0.89) (Fig. 2). Compared to the unvaccinated group, women who received the COVID-19 vaccines had similar rates of ongoing pregnancies or live birth (RR 1.00, 95% CI 0.97–1.03, *I*^2^ 10.7%) (Fig. 3).

## Discussion

We identified 21 studies reporting miscarriage or live birth/ongoing pregnancy outcomes among 149 685 women. Our results demonstrate no apparent increase in the risk of miscarriage among pregnant women who received the COVID-19 vaccines, which was consistent with the rate of miscarriage in the general population before the pandemic (Quenby *et al.*, 2021). Compared to unvaccinated women, those who received the vaccine had a slightly higher risk of miscarriage, though this was not statistically significant. This trend could be explained by several confounders, such as population socio-economics, baseline risk factors (e.g. recurrent pregnancy loss), co-morbidities, and access to healthcare services, which were observed in cohort studies evaluating third-trimester pregnancy outcomes among vaccinated women (Fell *et al.*, 2022; Magnus *et al.*, 2022). There was no significant difference in the RR of live birth or ongoing pregnancy among women who received COVID-19 vaccination compared to those who did not receive a vaccine.

**Figure 4.**
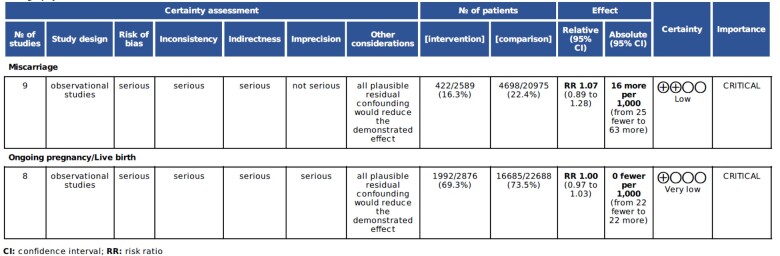
**GRADE evidence assessment table for the risk of miscarriage and ongoing pregnancy/live birth among pregnancy women who received COVID-10 vaccine**.

Overall, the certainty in the pooled evidence was low (Fig. 4) due to serious concerns about the consistency, precision and directness of our synthesized effect estimate. Given the high heterogeneity across included studies, our results should be interpreted with caution pending larger well-powered controlled studies.

### Strengths and limitations

We present a systematic review that employed a prospectively registered protocol and reported as per established guidelines, therefore offering a comprehensive assessment of the literature on the safety of COVID-19 vaccines in pregnancy. Only about half of the included studies had appropriately matched controls which limited our ability to generate a RR with accurate confidence intervals. Still, we reported narratively on all included studies and generated a weighted average to estimate the overall proportion of miscarriage and ongoing pregnancy or live birth among vaccinated pregnant women.

We included studies from various countries including data from large regulatory randomized trials that were used to licence the use of COVID-19 vaccine in the general population. However, as pregnant women were excluded from these trials at the time of randomization, the evidence included in this review is mainly observational with high level of heterogeneity. Several factors could explain this heterogeneity including variation in study design and patient characteristics, and the high risk of bias across included studies. This limits the generalizability of our meta-analysis and highlights the need for better quality primary studies involving pregnant women.

The majority of the included studies practiced suboptimal and varied outcome reporting which limited our ability to synthesize high-quality evidence, as reflected in our GRADE assessment (Fig. 4). This reduced the certainty of our pooled estimates, especially since other important pregnancy outcomes, e.g. stillbirth and ectopic pregnancy, were not reported.

While we reported a relatively low miscarriage rate (9%) across a large cohort (n = 123 184), our pooled rate offers a limited snapshot assessment over a short period of time and therefore should be interpreted with caution. Clearly, several factors could influence the overall miscarriage rate during the pandemic such as ethnicity, mode of conception, and access to maternity services during lockdown periods (García-Enguídanos *et al.*, 2002).

As most of these studies focused on short snapshot assessment of COVID-19 vaccine safety, the majority reported on the combined outcome of ongoing pregnancy or live birth. Clearly, this outcome does not offer an accurate assessment of long-term reproductive outcomes as not all ongoing pregnancies captured will yield a live birth. Still, we chose to report on this outcome to provide an accurate summary of the current available literature, assess the knowledge gap, and make recommendations to improve the quality of future research.

We planned to perform meta-regression and subgroup analysis to evaluate and adjust for important confounders such as patient characteristics, vaccine types (e.g. mRNA versus vector), and the number of vaccine boosters. However, we were unable to produce these additional analyses due to poor reporting across included studies (Table I). Other important effect modifiers that were also poorly reported included patient age group, method of conception, multiple pregnancy, and the impact across first versus second-trimester miscarriage. Standardized outcome reporting is therefore essential to improve the quality of future evidence synthesis particularly to facilitate patient-level data analyses.

### Implications for clinical practice

The COVID-19 pandemic introduced unprecedented challenges with enduring humanitarian and economic crises that are still unfolding (Spinelli and Pellino, 2020). In addition to its high virality, rapid mutations, and lack of curative treatments, a key challenge in controlling the COVID-19 virus was the role of mass media misinformation that often undermined efforts to promote key prevention strategies like mask-wearing, social distancing, and vaccination (Roozenbeek *et al.*, 2020; Loomba *et al.*, 2021).

Generally, concerns about the safety of vaccines in pregnancy could be attributed to the generic immunological and inflammatory response that could impact foetal implantation and embryogenesis (Arora and Lakshmi, 2021; Moodley *et al.*, 2021). However, in the case of COVID-19 mRNA vaccines, there were concerns disseminated on social media platforms claiming higher risk of miscarriage due to the formation of antibodies that could cross the placenta and bind to the spike protein called syncytin-1, a critical protein in the formation of the syncytiotrophoblast layer of the human placenta and embryogenesis (Blake Evans *et al.*, 2021). Several studies have came out since to disprove these claims with no evidence from immunological studies to support such interaction (Moodley *et al.*, 2021). Our findings further support the lack of harmful evidence pending larger, better-quality studies at a population level.

Considering the increased risk of miscarriage and other adverse pregnancy outcomes associated with COVID-19 infection in pregnancy (Stock *et al.*, 2022), vaccines play a vital role to minimize the impact of this disease on pregnant women and their offspring (Arora and Lakshmi, 2021; Moodley *et al.*, 2021). Ideally, the risks of vaccination should be evaluated considering the patient’s current medical health, risk profile for COVID-19 morbidity, and past adverse reactions or febrile illnesses to previous vaccinations. Vaccinations in the first-trimester could pose some risks of high immunogenicity and inflammation from a febrile illness to the foetus; especially in patients who have few or no risk factors for serious morbidity should they contract COVID-19. However, the merits of avoiding COVID-19 vaccination in the first trimester in favour of the pre-conception period or the second trimester remain unclear and further research is needed.

Available COVID-19 vaccines seem to have high immunogenicity and reactogenicity (Gray *et al.*, 2021), often associated with a systemic inflammatory process manifesting with headache, myalgia, chills, and fever (Shimabukuro *et al.*, 2021). Pregnant women receiving COVID-19 vaccines reported a higher incidence of systemic fever after the second dose compared to non-pregnant women (Gray *et al.*, 2021). Fever in early pregnancy and during embryogenesis may be a teratogenic phenomenon and this may increase the risk of miscarriage especially in the first trimester or among those with more severe vaccine side effects (Graham *et al.*, 1998; Dreier*et al.*, 2014). We were unable to explore the optimal timing to provide COVID-19 vaccines in pregnancy and whether such side effects could have a differential impact on first versus second-trimester pregnancies.

As the rate of re-infection with new mutations of the COVID-19 virus is increasing progressively (Jain *et al.*, 2021), there is a need to evaluate the optimal timing to provide COVID-19 vaccines for both *de novo* and booster immunity. This is particularly relevant to high-risk women planning a pregnancy such as those with chronic disease or those undergoing assisted conception (Han *et al.*, 2022).

### Future research

There is a critical need to evaluate the short and long-term safety and effectiveness outcomes of the different COVID-19 vaccines on pregnant women and their offspring. As the use of different COVID-19 vaccines grows (mRNA versus vector vaccines), large prospective cohorts with appropriately matched controls are needed to evaluate the effectiveness and safety of the different COVID-19 vaccination programmes in reducing the reported risks of adverse maternal and neonatal outcomes (Wei *et al.*, 2021).

Several studies have identified binding and neutralizing antibody titres for COVID-19 in infant cord blood and the breast milk of lactating vaccinated women. This could suggest long-lasting protective immunity that might help to reduce the risk of re-infection or severe disease among this vulnerable cohort (Fell *et al.*, 2022; Goldshtein *et al.*, 2022; Magnus *et al.*, 2022). However, more epidemiological and translational studies are needed to evaluate the long-term health outcomes among both mothers and offspring post vaccine exposure.

We encountered a high degree of varied outcome reporting which significantly hindered effective evidence synthesis. Future studies should adopt standardized reporting of core outcomes as per published core sets for miscarriage, fertility and pregnancy to enable more efficient evidence synthesis and reduce research wastage (Smith *et al.*, 2017; Duffy *et al.*, 2020b, 2020a).

## Conclusions

COVID-19 vaccines are not associated with an increased risk of miscarriage or decreased rates of ongoing pregnancy or live birth rates among women of reproductive age. The current evidence remains limited and larger population studies are needed to evaluate the effectiveness and safety of COVID-19 vaccines in pregnancy.

## Supplementary Material

dead036_Supplementary_Data_File_S1

dead036_Supplementary_Figure_S1

dead036_Supplementary_Figure_S2

dead036_Supplementary_Table_SI

## Data Availability

All data generated or analysed during this study are included in this published article and its Supplementary Information files.
