# Supplementary material for: The risk of miscarriage following COVID-19 vaccination: a systematic review and meta-analysis
Source: Hum Reprod. 2023 Feb 16;38(5):840–52. doi: 10.1093/humrep/dead036 (PMC10152171; doi:10.1093/humrep/dead036)
Supplement: dead036_Supplementary_Data_File_S1 [file dead036_supplementary_data_file_s1.pdf]

Human Reproduction, pp. 1–1, 2023
https://doi.org/10.1093/humrep/dead036

human reproduction

SUPPLEMENTARY DATA

Supplementary Data File S1

Search strategy to identify primary studies that evaluated the risk of miscarriage among pregnant women who received COVID-19 vaccine.

1 exp COVID-19 Vaccines/

2 (Pfizer-BioNTech or Comirnaty or Moderna or Spikevax or (Johnson adj2 Johnson) or Janssen or AstraZeneca or AZD1222 or Vaxzevria or Covishield or ChAdOx1 or BBIBP-CorV or BIBP or Sinopharm or Sputnik* or Gam- COVID-Vac-8 or Sputnik light or CoronaVac or Sinovac or Dream vaccin*).mp.

3 1 or 2

4 exp SARS-CoV-2/

5 exp COVID-19/

6 (COVID 19 or Corona* or 2019-n* or novel CoV or sarscov2 or 2019nCoV or nCOV or COVID-19 or SARS-CoV-2 or txid2697049).mp.

7 4 or 5 or 6

8 exp Immunization/

9 exp Vaccines/

10 (Vaccin* or Immuni* or injection* or Inoculat* or boost*).mp.

11 8 or 9 or 10

12 7 and 11

13 3 or 12

14 exp Pregnancy Outcome/

15 Pregnancy Complications/

16 exp Pregnancy, High-Risk/

17 exp abortion, spontaneous/

18 ((f? etal or f? etus*) adj3 (death* or die* or dead or decease*)) or ((recur* or habitual or spontaneous or tubal) adj2 (abort*)) or miscarr* or (pregnan* adj3 outcome) or (pregnan* adj3 los*)

19 14 or 15 or 16 or 17 or 18

24 13 and 19
