# Supplementary material for: The risk of miscarriage following COVID-19 vaccination: a systematic review and meta-analysis
Source: Hum Reprod. 2023 Feb 16;38(5):840–52. doi: 10.1093/humrep/dead036 (PMC10152171; doi:10.1093/humrep/dead036)
Supplement: dead036_Supplementary_Figure_S2 [file dead036_supplementary_figure_s2.pdf]

Human Reproduction, pp. 1–1, 2023
https://doi.org/10.1093/humrep/dead036

human reproduction

SUPPLEMENTARY DATA

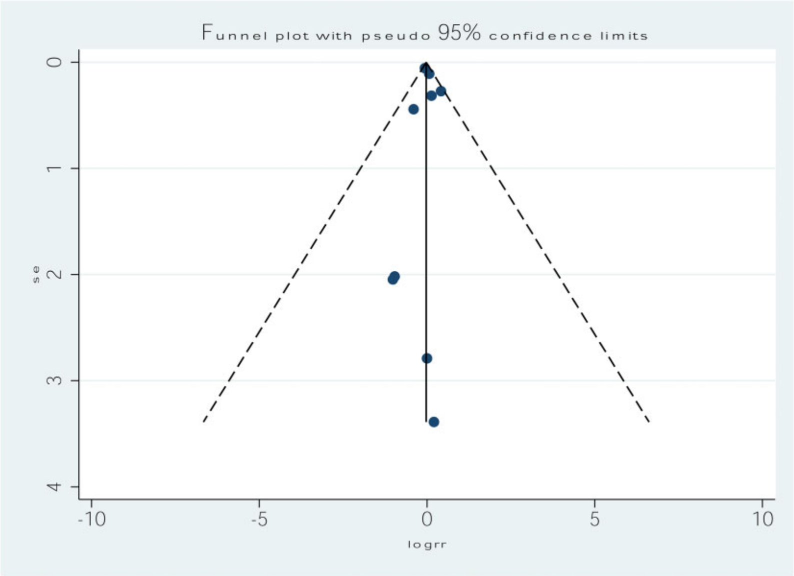

Supplementary Figures S2. **Funnel plot showing the variation in effect estimates by standard error across studies that evaluated the risk of miscarriage among pregnancy women who received COVID-19 vaccine.**
