# Supplementary material for: The risk of miscarriage following COVID-19 vaccination: a systematic review and meta-analysis
Source: Hum Reprod. 2023 Feb 16;38(5):840–52. doi: 10.1093/humrep/dead036 (PMC10152171; doi:10.1093/humrep/dead036)
Supplement: dead036_Supplementary_Table_SI [file dead036_supplementary_table_si.pdf]

Human Reproduction, pp. 1–3, 2023
https://doi.org/10.1093/humrep/dead036

human reproduction

SUPPLEMENTARY DATA

Supplementary Table SI **Summary of the characteristics and findings of included studies that evaluated the risk of miscarriage and ongoing pregnancy/live birth among pregnant women who received COVID-19 vaccine.**

Study	Settings	Participants	Outcomes
Aharon (2022)	Single-centre retrospective observational study of women who underwent controlled ovarian hyperstimulation for IVF or single euploid frozen-thawed embryo transfer.	Women who received two doses of Pfizer or Moderna COVID-19 vaccination at least 14 days prior to starting medication for controlled ovarian hyperstimulation or frozen-thawed embryo transfer cycle were included. A control group consisted of unvaccinated women undergoing controlled ovarian hyperstimulation or frozen-thawed embryo transfer cycles.	Higher parity, lower use of antagonist protocol, and higher use of the flare protocol was observed in the control group. No associations were observed between vaccinated and control groups with regard to fertilization rate (primary outcome), clinical pregnancy rate or rate of miscarriage.
Avraham (2022)	Retrospective observational study with data from two centres of women undergoing IVF treatment.	200 women who had received at least two doses of Pfizer COVID-19 vaccination were compared to 200 age-matched non-vaccinated controls.	The main outcome measures were the mean number of oocytes retrieved and clinical pregnancy rate. The mean number of oocytes retrieved and clinical pregnancy rate were similar among vaccinated women and non-vaccinated controls. Additionally, no difference was observed in fertilization rate, embryo quality or mean number of cryopreserved embryos between vaccinated and non-vaccinated controls.
Bleicher (2021)	Prospective observational cohort study where short-term pregnancy outcomes were assessed among vaccinated (Pfizer) and unvaccinated pregnant women through online questionnaires.	An initial questionnaire was shared via social media with responders being invited to complete a follow-up questionnaire after 1 month. Data collected included vaccination intentions, vaccination status, aspects of personal medical and obstetric history, and complications of their current pregnancy. The method by which pregnancy and miscarriage were confirmed was not defined.	432 women responded to the initial questionnaire, with the follow-up questionnaire receiving 326 responses. No significant differences in composite pregnancy complications, first trimester miscarriage, or other adverse obstetric outcomes were observed between vaccinated and unvaccinated groups.
Bookstein Peretz (2021)	Observational case-control study of pregnant women vaccinated with a two-dose Pfizer regimen aiming to assess the vaccines' immunogenicity, reactogenicity and impact on obstetric outcomes.	Outcomes among vaccinated pregnant participants were compared to a control group comprising age-matched non-pregnant vaccinated women. Pregnant participants were recruited via social media, and data was collected via serial questionnaires and blood sampling.	390 women returned the questionnaire and were included in the study alongside 260 control women. The method by which pregnancy and miscarriage were confirmed was not defined. Adverse obstetric outcomes were rare, and comparable to the general population. The Pfizer vaccine-induced humoral immunity in all vaccinated pregnant participants; however, levels of SARS-CoV-2 IgG were lower in pregnant women compared to non-pregnant controls.
Citu (2022)	Single-centre retrospective observational study of pregnant women in the first trimester who were either vaccinated or unvaccinated.	The method by which pregnancy and miscarriage were confirmed was not defined.	The risk of miscarriage after mRNA COVID-19 immunization iis commensurate with the predicted risk in non-vaccinated pregnant women.

(continued)

Supplementary Table SI Continued

Study	Settings	Participants	Outcomes
Favre (2022)	Swiss nationwide multicentre prospective cohort study	Pregnant women who received at least one dose of mRNA vaccine using the COVI-PREG registry.	Early and late spontaneous abortion was reported in 1/107 patient and 1/228 patient, respectively. No stillbirth was reported among 530 patients exposed with COVID-19 vaccines in pregnancy.
FDA—Janssen (2021)[a]	A large Phase 3 randomized, double-blinded placebo-controlled trial of a single dose of Ad26.COV2.S in ∼40 000 participants in the USA for assessment of safety, immunogenicity, and efficacy endpoints.	Participants who were pregnant or planned to conceive within 3 months of vaccine administration were excluded.	Subgroup analyses of adverse events identified eight reported pregnancies (four vaccine, four placebo). One miscarriage was reported in each arm respectively. The method by which pregnancy and miscarriage were confirmed was not defined.
FDA—Moderna (2020)[b]	A large Phase 3 randomized, double-blinded placebo-controlled trial of two 100 µg doses of mRNA-1273	30 400 participants in the USA for assessment of safety and efficacy endpoints. A negative pregnancy test was required prior to receiving study intervention.	There were no miscarriages in the vaccinated group and one miscarriage in the placebo group. The method by which pregnancy and miscarriage were confirmed was not defined. Subgroup analyses of adverse events identified 13 reported pregnancies (6 vaccine, 7 placebo).
FDA—Moderna (Booster) (2020)[c]	An open-label intervention study where participants who had previously received two 50 µg or 100 µg doses of mRNA-1273 received a 50 µg booster dose of mRNA-1273.	343 participants in the USA were enrolled to assess the safety and immunogenicity of the booster dose.	Subgroup analyses of adverse events reported 1 miscarriage 52 days after receiving booster dose, and subsequently conceived 115 days after the booster dose.
FDA—Pfizer (2020)[d]	A large global Phase 1/2/3 randomized, double-blinded placebo-controlled pivotal registration study of two 30 µg doses of BNT162b2 vaccine.	44 000 participants for assessment of safety, immunogenicity, and efficacy endpoints. A negative pregnancy test was required prior to receiving study intervention. With ∼18 800 participants in both control and vaccinated groups, a comparable number of pregnancies were observed (11 vaccine, 12 placebo).	There were no miscarriages in the vaccinated group and one miscarraige in the placebo group. A cumulative analysis of post-authorization adverse event reported 270 pregnancies, of which there were 23 miscarriages reported. The method by which pregnancy and miscarriage were confirmed was not defined.
Hillson (2021)	4 Phase 1/2/3 randomized, double-blinded placebo-controlled trials of two 0.5 ml doses of ChAdOx1 nCoV-19.	23 848 participants between April and November 2020 across UK, Brazil, and South Africa. A negative pregnancy test was required prior to receiving study intervention. Pregnancy outcome analysis set included 107 out of 9755 women of childbearing age who reported a pregnancy (72 vaccine, 35 control).	Miscarriage was defined as pregnancy loss before 23 weeks of gestation. The method by which pregnancy was confirmed was not defined. There were no evidence of an association between reduced fertility and vaccination, excluding Brazilian data, 11 miscarriages were reported (6 vaccine, 5 control),
Huang (2022)	Single-centre retrospective matched case-control study of women undergoing fresh IVF cycles.	Women vaccinated with two doses of Sinopharm or Sinovac comprised a case group and unvaccinated women comprised a control group. Cases and controls were matched using propensity scoring based on 14 covariates.	Similar outcomes including number of oocytes retrieved, good quality embryo-rate, clinical pregnancy rate, and biochemical pregnancy rate were observed between case and control groups.
Kachikis (2021)	Large online prospective cohort study of adults who were pregnant, lactating, or planning pregnancy at the time of COVID-19 vaccination. Participants were recruited online to the University of Washington COVID-19 Vaccine in Pregnancy and Lactation Registry via chain-referral and snowball sampling.	Data including participant demographics, vaccine side-effects, and outcome data were collected via questionnaires. The method by which pregnancy and miscarriage were confirmed was not defined. 17 525 participants were included, including 7809 participants who were pregnant at the time of their first vaccine (Pfizer, Moderna, or Janssen) dose and 6586 pregnant participants who had received a second vaccine dose at the time of data analysis.	6244 individuals remained pregnant, and 49 individuals reported miscarriage.

(continued)

Supplementary Table SI Continued

Study	Settings	Participants	Outcomes
Kharbanda (2021)	Observational case-control study of COVID-19 vaccination during pregnancy and spontaneous abortion using the Vaccine Safety Datalink.	A database collaboration between the Centres for Disease Control and Prevention and nine US health systems. The likelihood of receiving a COVID-19 vaccine in the 28 days prior to a spontaneous abortion were compared with the likelihood of receiving a COVID-19 vaccine in the 28 days prior to index dates for ongoing pregnancies. The method by which pregnancy and miscarriage were confirmed was not defined.	Spontaneous abortions did not have an increased odds of exposure to COVID-19 vaccination compared to ongoing pregnancies.
Magnus (2021)	Observational case-control study from Norwegian health registries using data on first trimester pregnancies and COVID-19 vaccination.	Odds ratios for COVID-19 vaccination in a period prior to miscarriage or ongoing pregnancy were estimated, adjusted for potential confounders and stratified according to number of vaccinations. The method by which pregnancy and miscarriage were confirmed was not defined.	No evidence of increased risk of miscarriage after COVID-19 vaccination among the study group.
Moro (2022)	Observational study of COVID-19 vaccination	Pregnant individuals using data from the Vaccine Adverse Event Reporting System (VAERS) across three different vaccines. The method by which pregnancy and miscarriage were confirmed was not defined.	Among 3462 reports involving pregnant women, there were 878 (25.4%) cases of miscarriage, 76 (2.2%) cases of preterm delivery, 62 (1.8%) cases of stillbirth, and 8 (0.2%) maternal deaths.
Nabila Arfah (2021)	Small prospective observational study of COVID-19 vaccination during pregnancy in	45 healthcare workers in Kedah, Malaysia who were found to be pregnant after vaccination. The method by which pregnancy and miscarriage were confirmed was not defined.	Five miscarriages were reported among this group, however due to limited data present the authors are unable to comment on the safety of COVID-19 vaccination safety during pregnancy.
Qiao (2021)	Observational study of COVID-19 vaccination	Pregnant individuals using data from the Brazilian surveillance information system for adverse events (SI-EAPV) across four different vaccines. The method by which pregnancy and miscarriage were confirmed was not defined.	Among 2486 reports involving pregnant and postpartum women, there were 59 (2.4%) reported cases of miscarriage, 13 (0.52%) cases of neonatal death, and 7 (0.28%) cases of preterm delivery
Trostle (2021)	Descriptive observational study	424 pregnant women who received at least one dose of mRNA COVID-19 vaccine in New York University Langone Health. The method by which pregnancy and miscarriage were confirmed was not defined.	9 miscarriages, 3 terminations, and 327 ongoing pregnancies were reported. No concerning trends were observed regarding birth outcomes among 85 women.
Wang (2022)	Retrospective observational study of cryopreserved embryo transfer cycles at a single tertiary centre in China.	Participants who had received two doses of inactivated COVID-19 vaccine were compared with unvaccinated controls.	Subgroups comprising those transferred cleavage-stage embryos and blastocysts were analysed, finding no differences in embryo implantation, clinical pregnancy or miscarriage rates between vaccinated and unvaccinated groups.
Zauche (2021)	Observational study of COVID-19 vaccination in pregnant individuals who received at least one dose of an mRNA COVID-19 vaccine either before conception or before 20 weeks gestation.	2456 pregnant women between 6 and 20 weeks gestation were identified from Centres for Disease Control and Prevention V-safe COVID-19 Pregnancy registry. The method by which pregnancy and miscarriage were confirmed was not defined. Life table methods were used to calculate the cumulative risk of miscarriage according to gestational week, with appropriate left truncation.	Sensitivity analysis after age standardization demonstrated the cumulative risk of miscarriage from 6 to <20 weeks gestation was 18.5% (95% CI, 16.1 to 20.8), which was within the expected risk range.

[a]https://www.fda.gov/media/146217/download.
[b]https://www.fda.gov/media/144585/download.
[c]https://www.fda.gov/media/152991/download.
[d]https://www.fda.gov/media/144246/download.
